# Supplementary material for: Including Phenotypic Causal Networks in Genome-Wide Association Studies Using Mixed Effects Structural Equation Models
Source: Front Genet. 2018 Oct 9;9:455. doi: 10.3389/fgene.2018.00455 (PMC6189326; doi:10.3389/fgene.2018.00455)
Supplement: Supplementary file 1 [file Table_1.docx]

***Supplementary Material***

**Including phenotypic causal networks in genome-wide association studies using mixed effects structural equation models**

**Running Head:** Structural equation modeling for association studies

Mehdi Momen^1^, Ahmad Ayatollahi Mehrgardi^1*^, Mahmoud Amiri Roudbar ^1^, Andreas Kranis^2^, Renan Mercuri Pinto^3,4^, Bruno D. Valente^4^, Gota Morota^5,^ Guilherme J. M. Rosa ^4,6^, Daniel Gianola^4,6,7^

^1^ Department of Animal Science, Faculty of Agriculture, Shahid Bahonar University of Kerman (SBUK), Kerman, Iran

^2^ Roslin Institute, University of Edinburgh, Midlothian, UK, EH25 9PS

^3^ Department of Exact Sciences, University of São Paulo - ESALQ, Piracicaba-SP, Brazil

^4^ Department of Animal Sciences, University of Wisconsin, Madison, WI, USA

^5^ Department of Animal and Poultry Sciences, Virginia Polytechnic Institute and State University, Blacksburg, VA, USA

^6^ Department of Biostatistics and Medical Informatics, University of Wisconsin, Madison, WI, USA

^7^ Department of Dairy Science, University of Wisconsin, Madison, WI, USA

*** Correspondence:**

Ahmad Ayatollahi Mehrgardi

mehrgardi@uk.ac.ir


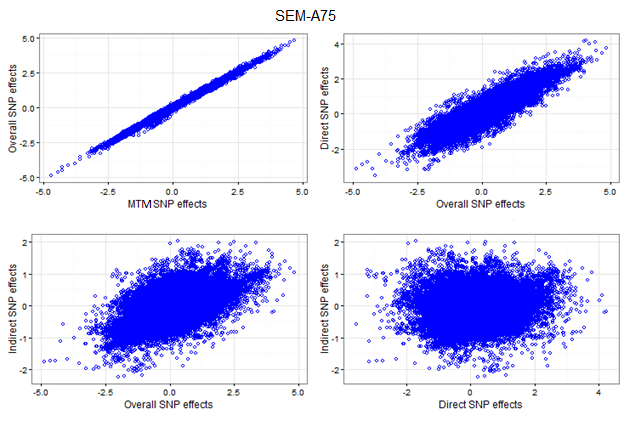


**Figure 1**. Scatter plots of comparing MTM-GWAS effects with 1) total effects and 2) decomposition of total effects into direct and indirect effects from SEM-A75. All decomposed effects are from the common BM🡪BW causal path.


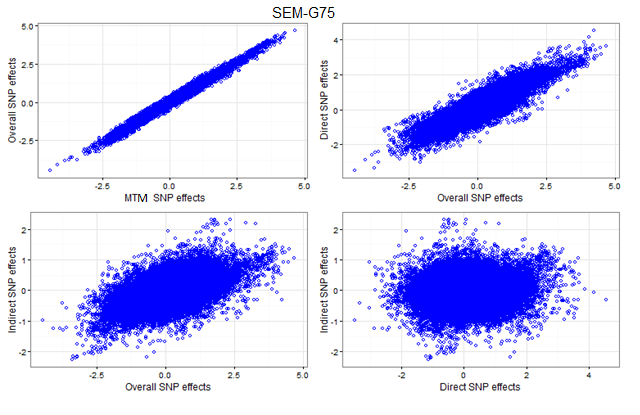


**Figure 2.** Scatter plots of comparing MTM-GWAS effects with 1) total effects and 2) decomposition of total effects into direct and indirect effects from SEM-G75. All decomposed effects are from the common BM🡪BW causal path.


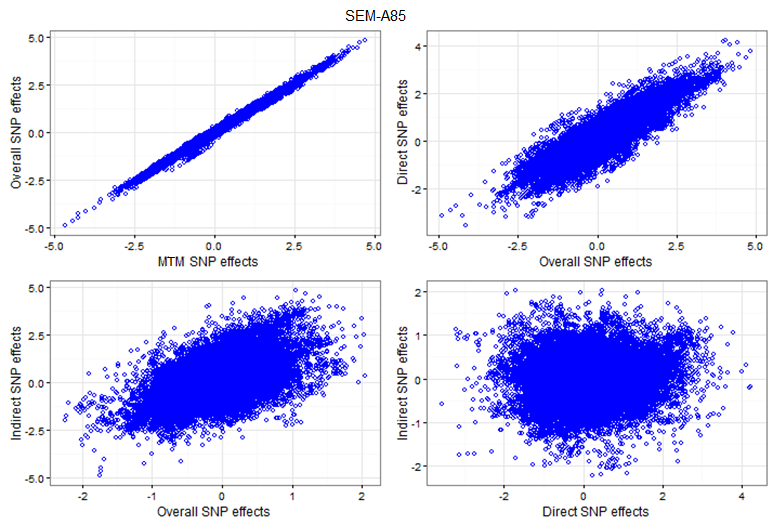


**Figure 3**. Scatter plots of comparing MTM-GWAS effects with 1) total effects and 2) decomposition of total effects into direct and indirect effects from SEM-A85. All decomposed effects are from the common BM🡪BW causal path.


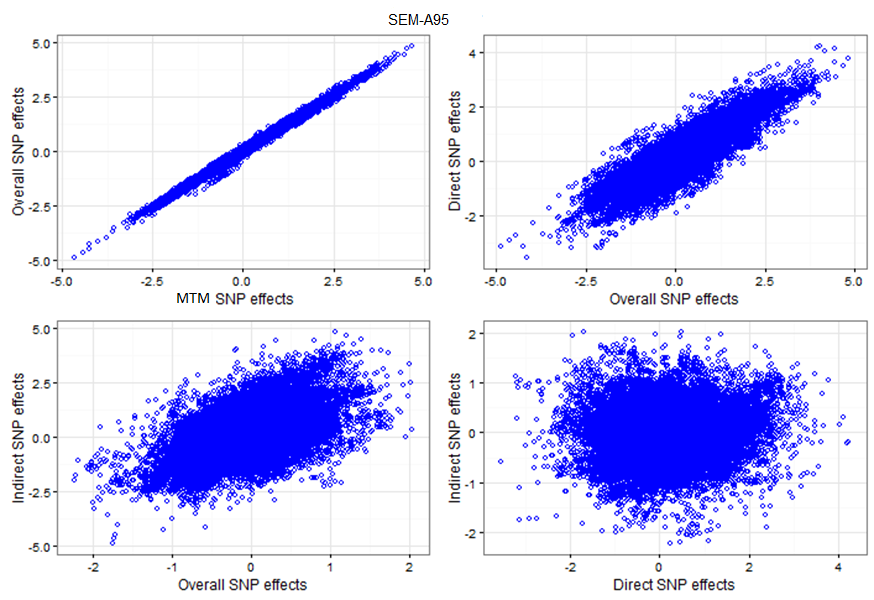


**Figure 4**. Scatter plots of comparing MTM-GWAS effects with 1) total effects and 2) decomposition of total effects into direct and indirect effects from SEM-A95. All decomposed effects are from the common BM🡪BW causal path.


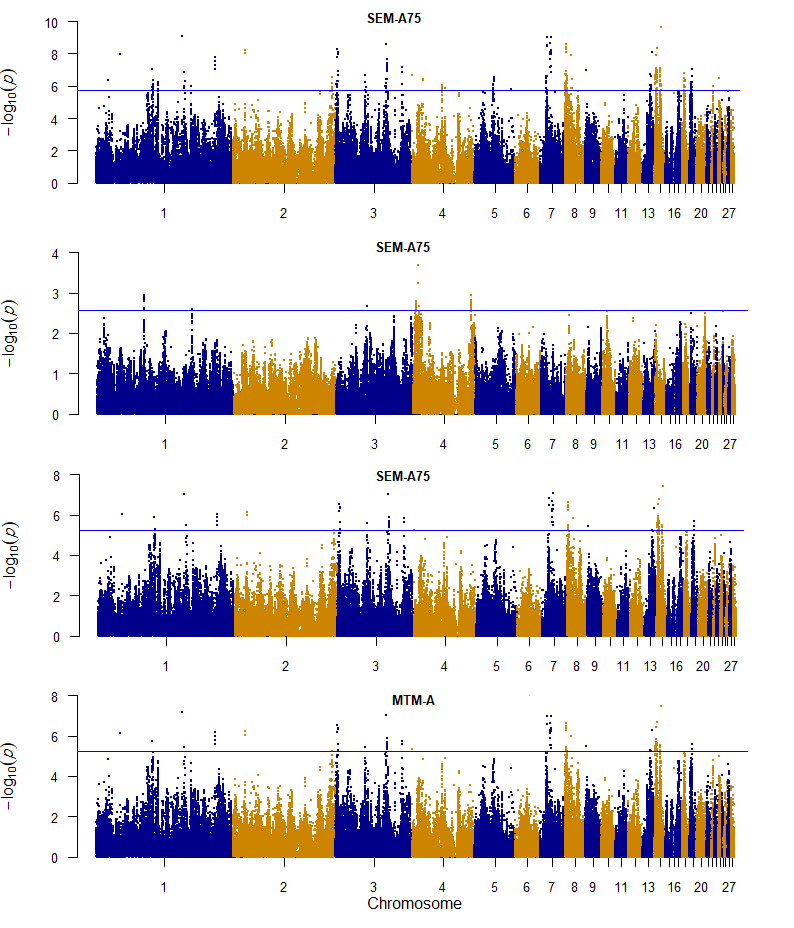


**Figure S5**. Manhattan plots showing –log_10_ (corrected p-value) of overall, direct, and indirect SNP effects using a full recursive model based on A matrix (SEM-A75) for house-hen egg production (HHP) and the total effects from multiple-trait (MTM-A). The horizontal blue line represent the threshold for false discovery rate of 5%.
